# Efficacy of conditioned autologous serum therapy (Orthokine®) on the dorsal root ganglion in patients with chronic radiculalgia: study protocol for a prospective randomized placebo-controlled double-blind clinical trial (RADISAC trial)

**DOI:** 10.1186/s13063-023-07787-y

**Published:** 2023-11-25

**Authors:** Marta Homs, Raimon Milà, Ricard Valdés, David Blay, Rosa Maria Borràs, David Parés

**Affiliations:** 1 Dexeus University Hospital, Sabino Arana 5-19, 08028 Barcelona, Spain; 2 Ramon Llull University, Pg St Gerbasi 43, 08022 Barcelona, Spain; 3 Barcelona, Spain; 4 Hospital Germans Trias I Pujol, Carretera del Canyet S/N, 08916 Badalona, Spain

**Keywords:** Lower limb radiculalgia, Pulsed radiofrequency, Dorsal root ganglion, Autologous conditioned serum

## Abstract

**Background:**

Pulsed radiofrequency (PRF) treatment on the dorsal root ganglion (DRG) has been proposed as a good option for the treatment of persistent radicular pain based on its effect of neuromodulation on neuropathic pain. Autologous conditioned serum (ACS) therapy is a conservative treatment based on the patient’s own blood. The aim of this manuscript is to develop a study protocol using ACS on the DRG as a target for its molecular modulation.

**Methods:**

We plan to conduct a randomized controlled study to compare the efficacy of PRF therapy plus ACS versus PRF therapy plus physiological saline 0.9% (PhS) on the DRG to reduce neuropathic pain in patients with persistent lower limb radiculalgia (LLR) and to contribute to the functional improvement and quality of life of these patients.

Study participants will include patients who meet study the inclusion/exclusion criteria. Eligible patients will be randomized in a 1:1 ratio to one of treatment with PRF plus ACS (experimental group) or PRF plus PhS (placebo group). The study group will consist of 70 patients (35 per group) who have experienced radicular pain symptoms for ≥ 6 months’ duration who have failed to respond to any therapy. Both groups will receive PRF on the DRG treatment before the injection of the sample (control or placebo). Patient assessments will occur at baseline, 1 month, 3 months, 6 months, and 12 months after therapy.

The primary efficacy outcome measure is Numeric Pain Rating Scale (NPRS) responders from baseline to 12 months of follow-up using validated minimal important change (MIC) thresholds. A reduction of ≥ 2 points in NPRS is considered a clinically significant pain relief.

The secondary efficacy outcome measure is the proportion of Oswestry Low Back Pain Disability Scale (ODS) responders from baseline to 12 months of follow-up in the experimental group (PRF plus ACS) versus the placebo group (PRF plus PhS). ODS responders are defined as those patients achieving the validated MIC of ≥ 10-point improvement in ODS from baseline to 12 months of follow-up as a clinically significant efficacy threshold.

**Discussion:**

This prospective, double-blind, randomized placebo-controlled study will provide level I evidence of the safety and effectiveness of ACS on neuropathic symptoms in LLR patients.

**Trial registration {2a}{2b}:**

EUDRACT number: 2021–005124-38. Validation date: 13 November 2021. Protocol version {3}: This manuscript presents the 2nd protocol version.

**Supplementary Information:**

The online version contains supplementary material available at 10.1186/s13063-023-07787-y.

## Introduction {6a}

Radicular pain is defined as pain perceived in an extremity or trunk wall caused by the activation of nociceptive afferent fibers from a spinal nerve (International Association for the Study of Pain, IASP) [1]. Due to the physiology of neuropathic pain itself, radicular pain is related to lesions that directly compromise the dorsal root ganglion (DRG) or directly compromise the spinal nerve and its roots by causing ischemia or inflammation of the axons [2, 3].

Lower limb radiculalgia (LLR) has an annual prevalence in the general population of 9.9% to 25%, which means that radicular pain is probably the most common form of neuropathic pain [4, 5]. LLR fully or partially resolves in 60% of patients within 12 weeks of symptom onset. However, in 20 to 30% of patients, the pain persists beyond 3 months and even beyond 12 months, and when this occurs, the prognosis is usually already unfavorable. In addition, the repercussion of neuropathic pain at the personal, social, and economic level of these patients is well known in the current literature. For all these reasons, it should not be underestimated that when it becomes chronic, neuropathic pain ceases to be a symptom and then becomes a disease [6–8].

Despite the availability of many treatments for LLR, the currently available evidence is insufficient for optimal therapy [9]. Conservative treatment of radicular pain combines pharmacological management with physiotherapy [10–14]. Interventional techniques with epidural steroid injections and surgery are then reserved for patients’ refractory to conservative treatment [11, 15], but some of them are unsuccessful.

The persistence of pain requires a neuro-histological and neuro-molecular consideration in order to find the adequate treatment targets. This is the reason why DRG is focused as a target for neuromodulation. DRG plays a key role in developing and maintaining radicular pain and its link to sensory disturbances. Apart from the neuron’s electrical potentials, the glia will drive an immune cascade of inflammatory mediators leading to peripheral and central sensitization. In addition, the hyperexcitability in afferent fibers is associated with changes in gene expression, in the ion channels themselves, leading to discharges, spontaneous bursts, which are the electrophysiological signature of neuropathic pain [16–18].

PRF therapy on the DRG has been proposed as a good option for the treatment of persistent radicular pain based on its power of neuromodulation on neuropathic pain [16, 19–30]. It is also minimally invasive, inexpensive, and simple to perform with few complications. The effect of PRF on neuropathic pain resides through different mechanisms, including the generation of heat and an electric field that induces changes in DRG neurons [30]. More than 120 articles dating back 15 years in terms of DRG PRFs are available in the current literature, giving in its best results an improvement of 29.5% at 2 months, and in 13.1% of the patients who improved, this improvement lasted up to 50% still at 12 months [27].

In order to be able to offer effective treatments for LLR, it is necessary open the field of research towards the cellular and molecular changes induced on the DRG. ACS therapy is a conservative treatment based on the patient’s own blood. A simple sample is taken from the patient, incubated and separated using a special device to produce a serum that is high in anti-inflammatory cytokines and growth factors. This serum is injected back into the patient in a series of 2 to 3 doses.

Described in multiple clinical trials, ACS therapy reduces pain and improves function, mobility, and quality of life. Patients are treated safely without too many clinically relevant side effects. None of their rival treatments such as corticosteroid, hyaluronic acid, or platelet recombinant plasma (PRP) are unrivaled in terms of the duration of their effectiveness in reducing pain [31–37]. When studying the pathophysiology at the molecular level, it is estimated that cytokines play a fundamental role in inflammatory processes, in the pathogenesis of joint degeneration, in spinal pathologies, in soft tissue degeneration, and in the immune system, being a factor in this key interleukin-1 [33–35, 38]. If, therefore, all these molecules are used in combination with natural concentration and autologous, better therapeutic effects can be obtained than exogenous drugs.

For all these reasons, in this study, it is proposed not only to treat patients with radicular pain from a neuromodulatory therapy with PRF but also to add an autologous molecular therapy for the optimization and duration of the clinical improvement of neuropathic radicular pain. In this way, it is intended to treat the DRG electromagnetically and neurochemically in order to achieve the modulation and total reversal of the neuronal malfunction that comprises neuropathic pain {6b}.

### Study design

#### Objectives {7}

The primary objective of the RADISAC study is to measure the effectiveness of the PRF therapy plus ACS on the DRG neuropathic pain in patients with persistent LLR.

The secondary objective is to evaluate the functionality, the quality of life, and the mood of patients during the first year after the treatment.

## Methods

This is a prospective, double-blind, randomized placebo-controlled trial designed to assess whether therapy with PRF + ACS on the DRG compared to PRF + PhS reduces neuropathic pain and its consequences, in patients with persistent LLR {8}.

In addition, this it to evaluate if the fact of adding ACS can give a greater advantage for the reduction of pain in these patients.

Taking into account that pulsed RDF is currently the interventional treatment with the best results to offer to patients with persistent chronic radiculalgia, it is the design of the study proposed to perform PRF therapy on the DRG for 8 min, 45 V of the root affected to all the patients included in the study. At the end of the PRF therapy, a 3-mL dose of ACS will be administered on the DRG to the patients of the experimental group and a 3-mL dose of 0.9% PhS will be administrated on the DRG to patients of the placebo group.

**Fig. 1:**
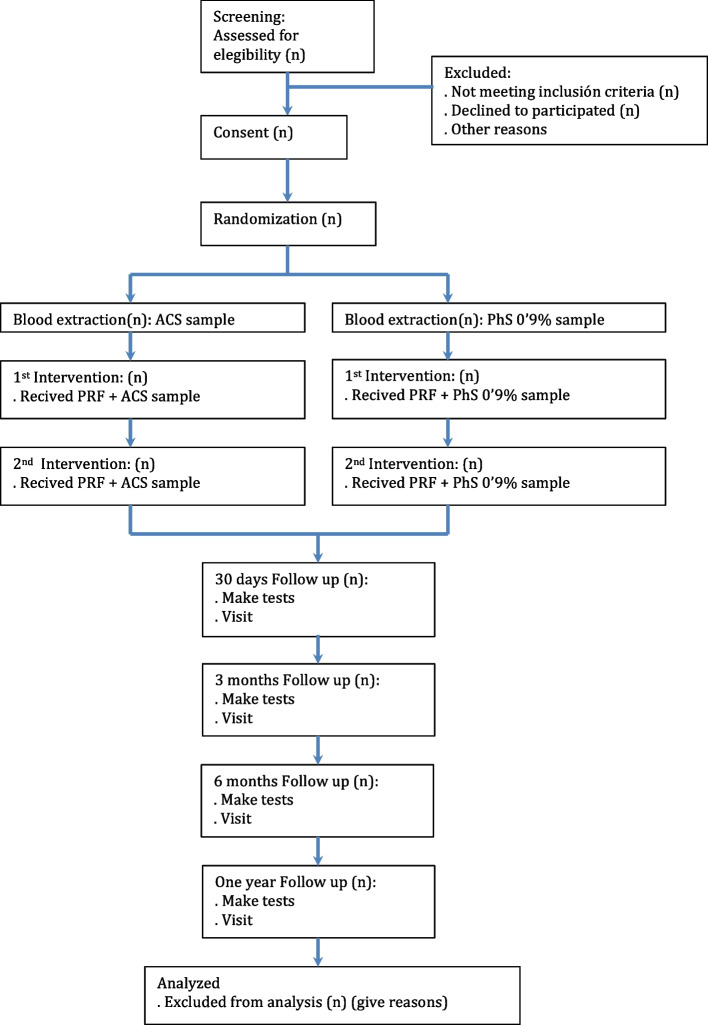
Study patient flow

A schematic patient flow diagram is presented in Fig. 1 as recommended by SPIRIT [39].

### Setting {9}

The project will be carried out in the facilities of the pain unit of Department of Anaesthesiology, Resuscitation and Pain Treatment at the Dexeus University Hospital (DUH) in Barcelona. The principal investigator is the first responsible of all the project and its development.

### Study population {10}

The study population proposed is 70 patients over 18 years of age, not illiterate, with radicular pain in the lower limb for more than 6 months. In addition, the symptomatic diagnosis screening criteria to confirm LLR will be used prior to the patient enrolment in the study. The inclusion and exclusion criteria are shown in Table 1.

**Table 1:**
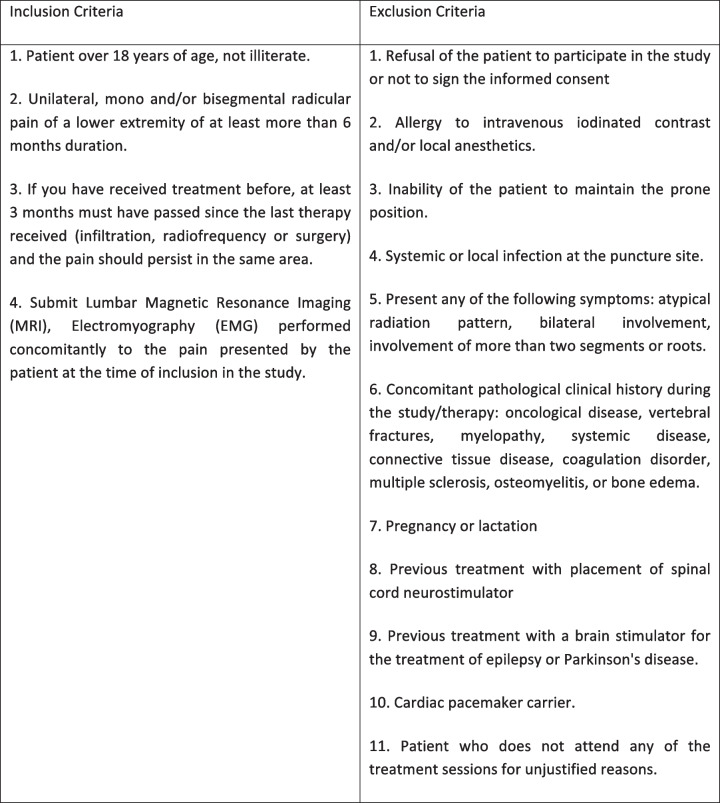
Inclusion and exclusion criteria

### Recruitment and informed consent

Both the main investigator and the other doctors of the pain unit will proceed to recruit patients within their usual clinical practice and according to inclusion and exclusion criteria {15}.

All the candidates will be informed in detail of the following information: the purpose of the study, interventions, benefits, possible risks, and corresponding responses. The patient accepts by signing the informed consent (IC) that he/she voluntarily participates in the study accepting that he/she may belong to the placebo or experimental group. They will sign the informed consent form voluntarily and will have the right to withdraw from the study at any time without any risk. The IC also includes a specific section on privacy consent and protection of personal data, according to the legal framework {26a} {26b} {6b}.

### Randomization {16a} {16b} {16c}

Randomization will be carried out using a simple randomization based on an equiprobable algorithm. According to randomization algorithm, a three-digit numerical code list will be generated with the R statistical software. It contains the three-digit and the assignation group. This list will be given to the laboratory and to the pharmacy service of the DUH, so only these both will know which of the groups is placebo and which of the groups is experimental. Only the part of the lists containing the 70 three-digit numerical code (not the assignation group) will be given to the main investigator. For each recruited patient, the main investigator will assign consecutively a three-digit code number and give to the patient a document with his name and the three-digit numerical code. When the patient will go to the laboratory, it will know if the patient belongs to the experimental group or the placebo group and proceed to prepare the samples. The principal investigator and the patients will be blinded for the remaining.

### Trial intervention and blinding {11a} {17a}

To comply with double-blind blinding, the following steps will be followed for the extraction and preparation of the ACS:

All patients will be scheduled 4 days before infiltration in the laboratory for blood collection and sample processing. Prior to extraction, patients will undergo the determinations based on the following documents: AEMPS REPORT/V1/23052013 and ROYAL DECREE 1088/2005. Given the laboratory will already know if the patient belongs to the experimental or placebo group, it will proceed to draw blood for the preparation of the ACS or not and store the syringes in the refrigerator at a temperature not exceeding – 18 °C.

All patients will undergo a blood draw for the preparation, or not, of the ACS, so they will not be able to know at any time which group they belong to. The syringes with the ACS or PhS samples are identical and sealed so that the main investigator will not be able to know to which group the patient belongs to. In addition, the way to evaluate the improvement or not of the pain will be through the self-assessment tests that the patient will answer individually at the control visits with what the main investigator will transcribe the results of the tests to the study database.

The day scheduled for infiltration, before performing the technique, adequate positioning of the patient, and proper monitoring in accordance with the protocol of the pain unit is mandatory. To perform the procedure, a transforaminal DRG approach technique will be performed under asepsis and antisepsis and under fluoroscopic vision. After checking the impedances (not higher than 450 ohms), the presence of sensory stimulation will be checked with a stimulation between 0.3 and 0.6 V and motor stimulation with a voltage at most double that is necessary to cause paresthesia. Finally, and once the injection of contrast is verified, PRF therapy will be carried out on the DRG (8 min, 45 V). Once the therapy is finished, the sample assigned to the patient will be injected.

Since most studies and clinical trials describe ACS therapy with 2 or 3 injections [31–37], separated in time by 7–15 days, the exact same procedure will be repeated 14 days after the first treatment.

Time schedule of enrolment, interventions, assessments, and visits for participants is shown in Table 2 (timeline) and Table 3 {13}.

**Table 2:**
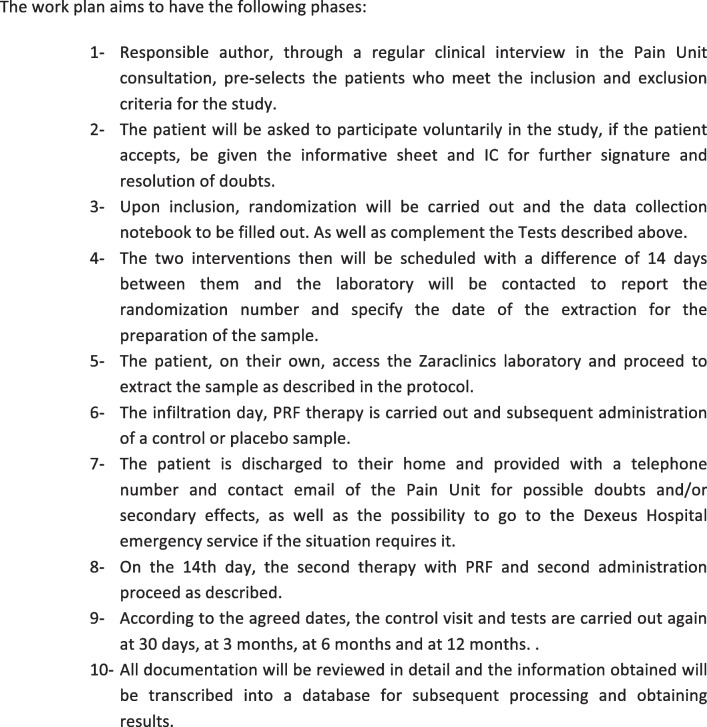
Timeline {13}

**Table 3:**
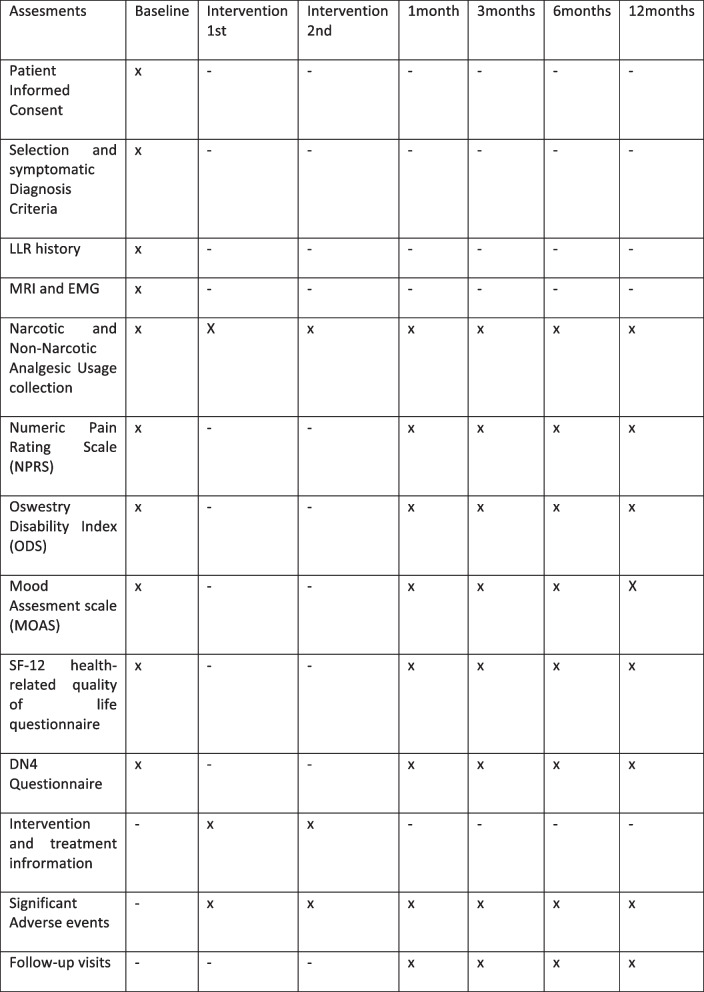
Schedule of assessments {13}

### Outcome measures and follow-up {12}

#### Primary outcome

The primary outcome will be level pain using Numeric Pain Rating Scale (NPRS). The NPRS is a segmented numeric version of the visual analog scale (VAS) in which a respondent selects a whole number (0–10 integers) that best reflects the intensity of his/her pain. A reduction of ≥ 2 points in NPRS is considered clinically significant pain relief [40]. This outcome will be measured at baseline at the moment of clinical interview and decision to recruitment and CI singed. This outcome will be measured again after the second intervention at 30 days, 3 months, 6 months, and 12 months after the intervention.

### Secondary outcomes

The secondary outcomes include the following: Oswestry Low Back Pain Disability Scale (ODS) [41–43], Scale for Mood Assessment (MOAS) or Mood Rating Scale (MRS) [in Spanish original name *Escala de Valoración del Estado de Ánimo (EVEA)*] [44], Quality of Life (SF 12) test [45–47], and *Douleur Neuropathique 4 questions* (DN4) scale [48, 49]. They will be measured at baseline and after 1 month, 3 months, 6 months, and 12 months of the intervention (Additional file 1: Annex I).

The total duration of the randomized placebo-controlled study is expected to be 36 months, including 24 months for patient recruitment and 12 months for final patient follow-up.

### Data collection {18a} {18b} {19} {27}

At baseline, patient demographics and prior failed therapies will be documented, including frequency, duration, type, and compliance, as well as magnetic resonance imagining (MRI) and electromyography (EMG) diagnostic tests. Documentation of analgesic use for the treatment of LLR will include frequency, dosage, and duration. All baseline and post-procedural follow-up points will include reported outcomes documented through completing the NPRS, ODS, MOAS, SF-12, and DN4 questionnaires and documentation of narcotic and non-narcotic analgesic medication usage. Device and procedure-related adverse events and all serious adverse events will be documented throughout the study period (Table 3).

The data collected will be stored in locked cabinets, and only the main researcher will have access to this information. Data will be recruited in a computer spreadsheet with password protection to ensure confidentiality. To ensure no error, the spreadsheet will be monthly monitored and audited by a researcher who is blind to the participants’ group allocation and has no conflict of interest.

All patients who leave the study for any reason will no longer have their data collected.

### Monitoring, safety, and quality control

The primary safety endpoint is the incidence of device- and/or procedure-related adverse events, which will be compared statistically between the randomized groups using proportions of patients experiencing such an event. The endpoint will be met if this event rate is not significantly greater with ACS than with PhS. All study-related adverse events will be monitored and reported, including seriousness, severity, treatment, and relationship to the study device/procedure. Adverse events collected in this study will be determined by the mine investigator to be specifically related to the products or procedures used in the 2 treatment groups or one that is determined to be a serious adverse event {22}.

To date, all published studies with ACS have shown high safety rates and no side effects for what ACS product itself represents. Even so, any intervention can lead to an adverse effect, so their monitoring will be an essential condition in this protocol {11b}.

Once the study has started, given that the inclusion of patients will be progressive over time as they are recruited, just in case there is a worsening of pain of more than 25% in more than 50% of the patients, the study would be reconsidered and the recruitment would be stopped to analyze the data and check if the worsening is in patients whom the ACS therapy was applied. If this were the case, the study would be definitively stopped and a report would be given to the laboratory. The author responsible for the study will carry out this audit if necessary. The main investigator together with the rest of the authors will make the decision to terminate the trial early {17b} {21a}.

Any other adverse effect as far as the interventions are concerned will be collected and analyzed (if necessary, the second intervention would be canceled, and the patient would be withdrawn from the study) {11b} {21b}.

The main investigator is the only person who will be in charge of follow-up with clinical interviews at 30 days, 3 months, 6 months, and 12 months. A schedule of visits will be established for each patient for their greater adherence to the study and not to delay follow-up. Apart from the following-up visit controls, the patients will provide from a telephone number of pain unit for any doubts, changing pain characteristics, or new interventions they will receive. In that way, it provides an improving of adherence to protocol of the study {11c}.

As far as the samples are concerned, both the laboratory responsible for the ACS and the pharmacy of DUH will have a record of the samples and their transport and temperature. Each sample will have a special label (study code, randomization number) and will be guarded by the pharmacy upon arrival from the laboratory and when it is withdrawn by the investigator on the day of its use for injection.

The study will be carried out in the facilities of the DUH, a document of suitability of the facilities signed by the head of the Department of Anaesthesiology and pain unit. In the same way, a commitment document is attached by the Hospital’s Pharmacy Department for the control and monitoring of the study. The main investigator will take the responsibility to monitoring of the study. She will coordinate with the people in the laboratory, pharmacy, and pain unit. There is also a thesis director who will meet monthly with the author responsible for the study and running the trial day-to-day to provide organizational support. In conclusion, since the day-to-day task falls on the main investigator, she will be responsible for any important protocol modification and technical aspects of the trial as well as data collection, outcome analyses, and results. She will be in charge of notifying the rest of authors as well as laboratory and pharmacy {5d} {21a} {25}.

### Sample size {14}

Based on the existing bibliography in relation to the treatment of pain by ACS treatment for other similar affections [32], it is estimated that accepting an alpha risk of 0.05 and a beta risk of 0.2 in a bilateral contrast, 35 subjects in the treatment group and 35 in the placebo group are needed to detect as statistically significant the difference between the proportion of patients with reduction pain of two points on the NPRS scale, which for the intervention group is expected to be 65%, and for the control group, it is expected to be 35%. A rate of loss to follow-up of 10% has been estimated.

### Statistical analysis {20a} {20b} {20c} {18b}

A descriptive analysis will be carried out for all the data collected. The normality of the data will be studied using the Kolmogorov–Smirnov and Shapiro–Wilk tests. For continuous quantitative variables, descriptions of central tendency and dispersion will be presented: mean and standard deviation respectively in case the variables follow a normal distribution or median and interquartile range for those variables that are not normally distributed. For categorical (qualitative) variables, frequencies and percentages will be presented. The main outcome (improvement > 2 points) will be tested using a generalized linear mixed model (GLMM). The GLMM model fit will include a between-subject factor and will also include a random effect for the time (baseline, 1st month, 3th month, 6th month, and 12th month) and the interaction between group and time variables. The inclusion of the interaction terms allows for a formal test between the groups over time. For the other variables, the mean effects of the interventions and the differences between groups and their respective 95% confidence intervals will be calculated using regression linear mixed models for repeated measures, which will incorporate terms for the treatment groups, time, and interaction terms. Treatment coefficients versus time interactions will be equivalent to the estimates of the differences between groups. All models will be adjusted for chance imbalances in outcome between groups at baseline. There will also be analyses of the covariates collected, including age, gender, and duration of symptoms. The analyses will follow the principles of intention to treat, and no interim analyses will be performed. No additional analysis will be performed. If a patient drops out of treatment, no additional outcome will be collected. The significance level will be set at 5%, and SPSS for Windows will be used for the statistical analysis.

The number of participants who complete the 12-month follow-up will be described by allocation; the study arms will be compared using chi-square tests and logistic regression to see if the attrition rate differs by arm and to compare baseline characteristics of participants who did and did not complete follow-up. Of those who complete follow-up, each variable will be examined for the presence of missing data, and if > 10% is observed for primary or secondary outcomes, then sensitivity analyses will be performed using complete case analysis or multiple imputation methods assuming data are missing at random (MAR). The MAR assumption indicates that the propensity for missingness does not depend on the unobserved outcome but rather is related to some other observed data.

### Pain management

Special care different from the patient’s own reality will not be applied during the course of the trial; that is intended to resemble the real clinical applicability of the treatment as much as possible. In those patients who take opioid and non-opioid analgesics and/or neuromodulators as usual, medication will be documented in the baseline and follow-up controls. Likewise, if a patient undergoes any infiltration or surgical intervention in the same area affected by the trial intervention, this patient should be excluded from the clinical trial algorithm, and their data may not be used in the results from the date of surgery or infiltration {11d}.

## Discussion

This study intends to adopt a rigorously designed and implemented randomized controlled method to evaluate the efficacy and safety of the administration of PRF therapy, combined with ACS, on the DRG to reduce neuropathic pain in patients with persistent LRP. The aim of this study is to explore a treatment with greater efficacy and fewer side effects for patients with persistent LLR, who have found pharmacologic therapy, corticoid injection therapy, or surgery ineffective. This treatment, if proven effective, may then be used as a complementary treatment to a more persistent pain.

A number of clinical studies show that ACS (Orthokine®) therapy reduces pain and improves function, mobility, and health-related quality of life. Patients can be treated safely and effectively, and no other injection therapy (corticosteroids, hyaluronic acid, PRP) rivals its long-term efficacy in osteoarthritis or musculoskeletal pathologies [31–37]. The use of ACS (Orthokine®) therapy may help to reduce the number of surgeries, the dosage and frequency of pain killing medications, and therefore medical costs in the medium and long term.

To date, only the randomized clinical trial by Becker et al. has shown superiority of ACS over corticosteroids in lumbar radicular pain (3 epidural injections) [32]. Godek et al. in 2016 also proposed a pilot study in which he observed pain improvement in 15 patients who were injected with ACS under ultrasound vision in the foramen affected by lumbar disc herniation [50]. Kumar et al. proposed 20 patients with unilateral lumbar radiculalgia in whom he applied between 1 and 3 injections of ACS in the epidural space; the patients improved their pain at 3 weeks, 3 months, and 6 months, but there was no control group in any of two studies [51].

In all cases, there were no undesirable or secondary effects due to ACS that made the studies reconsider in terms of safety. There is even a randomized pilot study also by Goni in which ACS injections were at cervical epidural level and in which pain improvement was obtained compared to methylprednisolone [52].

The data, daily regular use, and yearlong experience suggest ACS (Orthokine®) serum is an effective and well-tolerated alternative to other injection therapies.

As Kuffler explains [53], the trigger axon regeneration and Schwann cell proliferation may assist in reducing neuropathic pain. All molecules contained in ACS could give that trigger point. This study aims to highlight the importance of neuromodulation at the molecular level and how treatment with ACS (rich in cytokines, growth factors and especially interleukins) can give a very advantageous synergy to treatment LLR compared to using only corticosteroids or even other platelet-rich plasma serum.

## Conclusions

If the hypothesis proposed in the study is proven, a great advance will be made in the treatment of LLR and even further neuropathic pain. If there is an improvement in pain in these patients and this has repercussions on an improvement in their quality of life and functionality, we will not only be contributing to the clinical improvement of pain itself but also contributing to the emotional and psychological part of life of these patients, transforming pain into a minimal symptom for it to cease to be a disease.

Furthermore, as future therapies, it will give rise to a new gateway to future applications of the ACS as a molecular therapy on other types of neuropathic pain such as trigeminal neuropathy, peripheral neuropathy, and syndrome of the complex regional pain, among others.

### Trial status

The protocol version number: 2, 1 September 2021.

Date recruitment began: 1 February 2022.

Approximate date when recruitment will be completed: 1 February 2024.

## Supplementary Information

**Additional file 1.** Annex I

## Data Availability

Only the main investigator will have access to the final trial dataset, who signed a data confidentiality commitment letter {29}. In addition, the main investigator signed a commitment letter as well as a data confidentiality commitment letter performing functions as a clinical record reviewer {27}.

## Abbreviations

PRF: Pulsed radiofrequency
DRG: Dorsal root ganglion
ACS: Autologous conditioned serum
PhS: Physiological saline 0.9%
LLR: Lower limb radiculalgia
MIC: Minimal important change
NPRS: Numeric Pain Rating Scale
ODS: Oswestry Disability Scale
IASP: International Association for the Study of Pain
DUH: Dexeus University Hospital
IC: Informed consent
VAS: Visual analog scale
MOAS: Scale for Mood Assessment
MRS: Mood Rating Scale
EVEA: *Escala de Valoración del Estado de Ánimo*
SF-12: Quality of Life Test
DN4: Douleur Neuropathique 4 questions
MRI: Magnetic resonance imaging
EMG: Electromyography

## Declarations

### Provisions for post-trial care {30}

In terms of patient safety, the Department of Anaesthesiology, Resuscitation and Pain Treatment provides a civil liability policy to cover the procedures. The company Orthogen Lab Services GmbH also intends to cover the administration of the therapy within the clinical trial with its civil liability policy already used in previous clinical trials of 1 year of renewable duration year after year.
