# Supplementary material for: Efficacy of conditioned autologous serum therapy (Orthokine®) on the dorsal root ganglion in patients with chronic radiculalgia: study protocol for a prospective randomized placebo-controlled double-blind clinical trial (RADISAC trial)
Source: Trials. 2023 Nov 25;24:755. doi: 10.1186/s13063-023-07787-y (PMC10676602; doi:10.1186/s13063-023-07787-y)
Supplement: Supplementary file 1 — Additional file 1. Annex I [file 13063_2023_7787_MOESM1_ESM.pdf]

Annex I

Oswestry Low Back Pain Disability Scale: the Oswestry Low Back Pain Disability Questionnaire is an extremely important tool that researchers and disability evaluators use to measure a patient's permanent functional disability (41). The test is considered the 'gold standard' of low back functional outcome tools. The Oswestry Low Back Pain Disability Scale is a self-administered questionnaire, specific for low back pain, that measures limitations in daily activities. The development of the Oswestry low back pain disability scale was initiated in 1976 by John O'Brien with patients referred to a specialized clinic who had chronic low back pain and is the most widely used and recommended worldwide to measure disability due to low back pain (42). The questionnaire consists of 10 items addressing different aspects of function. Each item is scored from 0 to 5, with higher values representing greater disability. The total score, expressed as a percentage (from 0 to 100%), is obtained by adding the scores for each item divided by the maximum possible score multiplied by 100:

$$\text{Total score} = \frac{50 - (5 \times \text{number of unanswered item})}{sum\ of\ the\ socres\ of\ the\ answered\ items}\ x\ 100$$

High values describe greater functional limitations. Between 0-20%: minimal functional limitation; 20%-40%: moderate; 40%-60%: intense; 60%-80%: disability, and above 80%: maximum functional limitation. ODS responders are defined as those patients achieving the validated MIC of ≥ 10 point improvement in ODS from baseline to 12 follow-up as a clinically significant efficacy threshold. This outcome will be measured at baseline, after 1 month, 3 months, 6 months and 12 months after the intervention (43).

Scale for Mood Assesment (MOAS) or Mood Rating Scale (MRS) [in Spanish original name: *Escala de Valoración del Estado de Ánimo (EVEA)*] (44) was constructed by Sanz as an instrument to measure transient mood states in studies using procedures. It consists of a 16-item test, each one composed of a Likert-type graphic scale of 11 points (from 0 to 10), flanked by the words "not at all" (0) and "a lot" (10), which presents in its left margin a short statement describing a state of mind. All 16 phrases have the same construction; they all begin with the words “I feel” and continue with an adjective that represents a mood (eg, “I feel sad,” “I feel happy”). The MOAS aims to assess four moods: anxiety, anger-hostility, sadness-depression and joy. Each mood state is represented by four items with different adjectives which define a subscale, and all the items within each subscale are formulated in the same direction. This outcome will be measured at baseline, after 30 days, 3 months, 6 months and 12 months after the intervention.

Quality of life (SF 12): SF-12 health-related quality of life questionnaire. Composed of twelve items, whose purpose is to provide an easy-to-apply instrument to assess the degree of well-being and functional capacity of people over 14 years of age, defining a positive and negative state of physical and mental health, through eight dimensions (physical function, physical role, body pain, mental health, general health, vitality, social function, and emotional role) (45-47).

The response options form Likert-type scales (where the number of options varies from three to six points, depending on the item), which assess the intensity and/or frequency of the person's health status. The score ranges from 0 to 100, with a higher score implying a better health-related quality of life. Research using the twelve items of the SF has verified that this instrument is a valid and reliable measure, finding internal consistency estimates greater than 0.70 and significant correlations between the versions of the scale. This outcome will be measured at baseline, after 30 days, 3 months, 6 months and 12 months after the intervention.

DN4 scale: The DN4 scale is a 10-question test on pain with neuropathic characteristics, all of which have a yes/no score. The answer "yes" adds a point and the answer "no" does not add a point. The final result from 0 to 10 gives an idea of how much neuropathic component the pain presented by the patient has (48,49). This outcome will be measured at baseline, after 30 days, 3 months, 6 months and 12 months after the intervention.
